# Reusable Client-Side JavaScript Modules for Immersive Web-Based Real-Time Collaborative Neuroimage Visualization

**DOI:** 10.3389/fninf.2017.00032

**Published:** 2017-05-01

**Authors:** Jorge L. Bernal-Rusiel, Nicolas Rannou, Randy L. Gollub, Steve Pieper, Shawn Murphy, Richard Robertson, Patricia E. Grant, Rudolph Pienaar

**Affiliations:** ^1^Fetal-Neonatal Neuroimaging and Developmental Science Center, Boston Children's HospitalBoston, MA, USA; ^2^Eunate Technology S.L.Sopela, Spain; ^3^Department of Radiology, Massachusetts General HospitalBoston, MA, USA; ^4^Department of Psychiatry, Massachusetts General HospitalBoston, MA, USA; ^5^Harvard Medical SchoolBoston, MA, USA; ^6^Isomics Inc.Cambridge, MA, USA; ^7^Surgical Planning Laboratory, Brigham and Women's HospitalBoston, MA, USA; ^8^Department of Neurology, Massachusetts General HospitalBoston, MA, USA; ^9^Laboratory of Computer Science, Massachusetts General HospitalBoston, MA, USA; ^10^Department of Radiology, Boston Children's HospitalBoston, MA, USA

**Keywords:** collaborative visualization, interactive visualization, real-time collaboration, neuroimaging, HTML5, web services, telemedicine, Google Drive

## Abstract

In this paper we present a web-based software solution to the problem of implementing real-time collaborative neuroimage visualization. In both clinical and research settings, simple and powerful access to imaging technologies across multiple devices is becoming increasingly useful. Prior technical solutions have used a server-side rendering and push-to-client model wherein only the server has the full image dataset. We propose a rich client solution in which each client has all the data and uses the Google Drive Realtime API for state synchronization. We have developed a small set of reusable client-side object-oriented JavaScript modules that make use of the XTK toolkit, a popular open-source JavaScript library also developed by our team, for the in-browser rendering and visualization of brain image volumes. Efficient realtime communication among the remote instances is achieved by using just a small JSON object, comprising a representation of the XTK image renderers' state, as the Google Drive Realtime collaborative data model. The developed open-source JavaScript modules have already been instantiated in a web-app called *MedView*, a distributed collaborative neuroimage visualization application that is delivered to the users over the web without requiring the installation of any extra software or browser plugin. This responsive application allows multiple physically distant physicians or researchers to cooperate in real time to reach a diagnosis or scientific conclusion. It also serves as a proof of concept for the capabilities of the presented technological solution.

## 1. Introduction

Diagnosis in complex medical disorders as well as imaging research can benefit from cooperative visualization and analysis of the same image volume by more than one physician or researcher at the same time in a session that shares control and events between all parties. The viewing parties often may not be located at the same physical location but are connected via some data network and their geographical separation can span different cities or even different countries. These location constraints together with the need for real-time interactions on the image data between participants calls for the development of the so-called collaborative image visualization systems (CIVS). In medical diagnosis, these types of systems form an important sub-area of Telemedicine (Manssour and Dal Sasso Freitas, 2000). CIVS can be seen as a type of distributed software system that attempts to provide simultaneous visualization of shared image data and automatic synchronization of user-data interactions among users working on physically remote computational entities (desktop computers, mobile devices, etc). This synchronization is difficult to achieve in real time as it usually has to be carried out over the Internet without assuming any specific network topology and latency or a predefined number of connected users. Furthermore, user accessibility and visualization synchronization are both affected by the fact that the remote computational entities can have quite different hardware architectures and operating system platforms. In addition CIVS are expected to provide a user-friendly homogeneous-across-platforms interface and to require minimal user technological skills for their installation and usage.

Several attempts to implement CIVS have been reported over the last two decades. Early solutions mainly ran on UNIX platforms because of the built-in network and security features. These remote visualization instances were interconnected using middleware technologies for distributed systems such as Common Object Request Broker Architecture (CORBA) or Remote Method Invocation (RMI) (Anupam et al., 1994; Forslund et al., 1996, 1998; Coleman et al., 1997). Cross-platform solutions began to appear around the turn of the century using java to implement the client-side software (Manssour and Dal Sasso Freitas, 2000). However, these technologies lack the desirable loose coupling between clients and servers and provide an unnecessary complex application programming interface (API) among other technical and cost issues (Gokhale et al., 2002; Henning, 2006).

The shift to full web-based solutions has occurred somewhat haphazardly since the early 2000s (Eckersley et al., 2003; Millan and Yunda, 2014; Sherif et al., 2014; Wood et al., 2014) and closely tracked the increasing rise and power of the web browser as a middleware platform and the expressiveness of the JavaScript programming language. Web-based solutions are especially appealing as they do not require the installation of any client-side software other than a standard web browser which enhances accessibility and usability. But as with the previous technologies in most cases collaboration only meant data sharing with no realtime interactivity between participants. Alternatively, when simultaneous visualization was required only a single user (the collaboration owner) could have direct control over the visualization parameters and the other users were limited to passive viewing of results using various streaming technologies. As such, real-time synchronization among physically remote highly interactive clients over the wide Internet has not been readily available.

Recently some web-based CIVS have been implemented that attempt to achieve real-time synchronization by rendering the image volumes on the server side and sending a representation of the visualization to each collaborator's web browser as a series of 2D images or streaming video (mainly JPEG, PNG, or MPEG formats) (Kaspar et al., 2010, 2013). However, this server-side rendering technique is not suitable for the so-called Fully-Shared-Control real-time CIVS in which all the collaborators have control over the parameters associated with a given interactive visualization (e.g., window leveling of the currently rendered image volume slice) (Manssour and Dal Sasso Freitas, 2000). The main reason for this is that it requires continuously sending relatively heavy data over the network after each single user-data interaction that modifies the visualization parameters. This makes the application not only highly sensitive to user-specific network latency but doesn't scale well when the number of concurrent users increases. Therefore, a distributed client-side rendering approach would be preferable for fast real-time all-users interactivity.

New advances in core web application technologies such as the modern web browsers' universal support of ECMAScript 5 (and 6), CSS3 and HTML5 APIs have made it much more feasible to implement efficient graphical image volume rendering and visualization as well as real-time communication purely in client-side JavaScript (Mwalongo et al., 2016). Indeed a few powerful client-side JavaScript libraries that can perform in-browser rendering and visualization of 3D neuroimage volumes have already emerged and are freely available as open-source projects (Sherif et al., 2015). In particular, in this paper we make use of the popular XTK toolkit which was developed by our team at the Fetal-Neonatal Neuroimaging and Developmental Science Center, Boston Childrens Hospital^1^ and can be freely downloaded from the web^2^ (Haehn et al., 2014).

Despite the ready availability of client-side JavaScript rendering libraries we only know of a single attempt at implementing a web-based real-time CIVS based on a client-side rendering and visualization approach. The Slice:Drop^3^ application previously developed by our team provides a solution based on the XTK toolkit and the Dropbox API (Haehn, 2013). However, Slice:Drop was developed mostly as a prototypical concept showcase and is less modular and difficult to reuse in other applications. In addition, Slice:Drop uses third party services provided by Dropbox and early implementations required data to be publicly shared without any restrictions which may lead to undesirable data leaking to non-intended Internet users.

In this article we propose a new web-based technological solution (in many ways a logical successor to Slice:Drop) to the problem of implementing efficient real-time collaborative neuroimage visualization. As with Slice:Drop, here we adopt a client-side rendering and visualization approach based on the XTK toolkit. We propose a portable viewing application that can easily be embedded into larger systems, such as ChRIS [also developed at Boston Children's Hospital (Pienaar et al., 2015)]. Real-time synchronization and communication among remote visualization instances is then managed through the Google Drive Realtime API. We provide a small set of reusable client-side object-oriented JavaScript modules named viewerjs^4^, fmjs^5^, and gcjs^6^ that are freely accessible as open source software from our Github organization repositories^7^. These JavaScript modules have already been instantiated in the implementation of a distributed collaborative neuroimage visualization application called *MedView* that is delivered to the users over the web without requiring the installation of any extra software or browser plugin. The source code of this application and a built and deployed version are also hosted in our Github repositories. It allows multiple physically distant physicians or researchers to cooperate in real-time to reach a diagnosis or scientific conclusion.

## 2. Materials and methods

### 2.1. Scope

The *MedView* application described in this paper provides a web-based viewer for common medical image formats. When clients visit the *MedView* website^8^, the required functionality is downloaded into, and executed by, the browser. Any medical image data that is “dropped" into the browser (or navigated to from the file picker dialog) stays in the local client context, i.e., no data is uploaded out to the web or to some remote website. All data remains local—only the viewer source code in the form of Javascript programming is fetched remotely.

In addition to image rendering, *MedView* provides for real-time collaboration and sharing of a common image cursor between all participants in a collaborative session.

### 2.2. Client-side rendering and visualization

**Figure 1:**
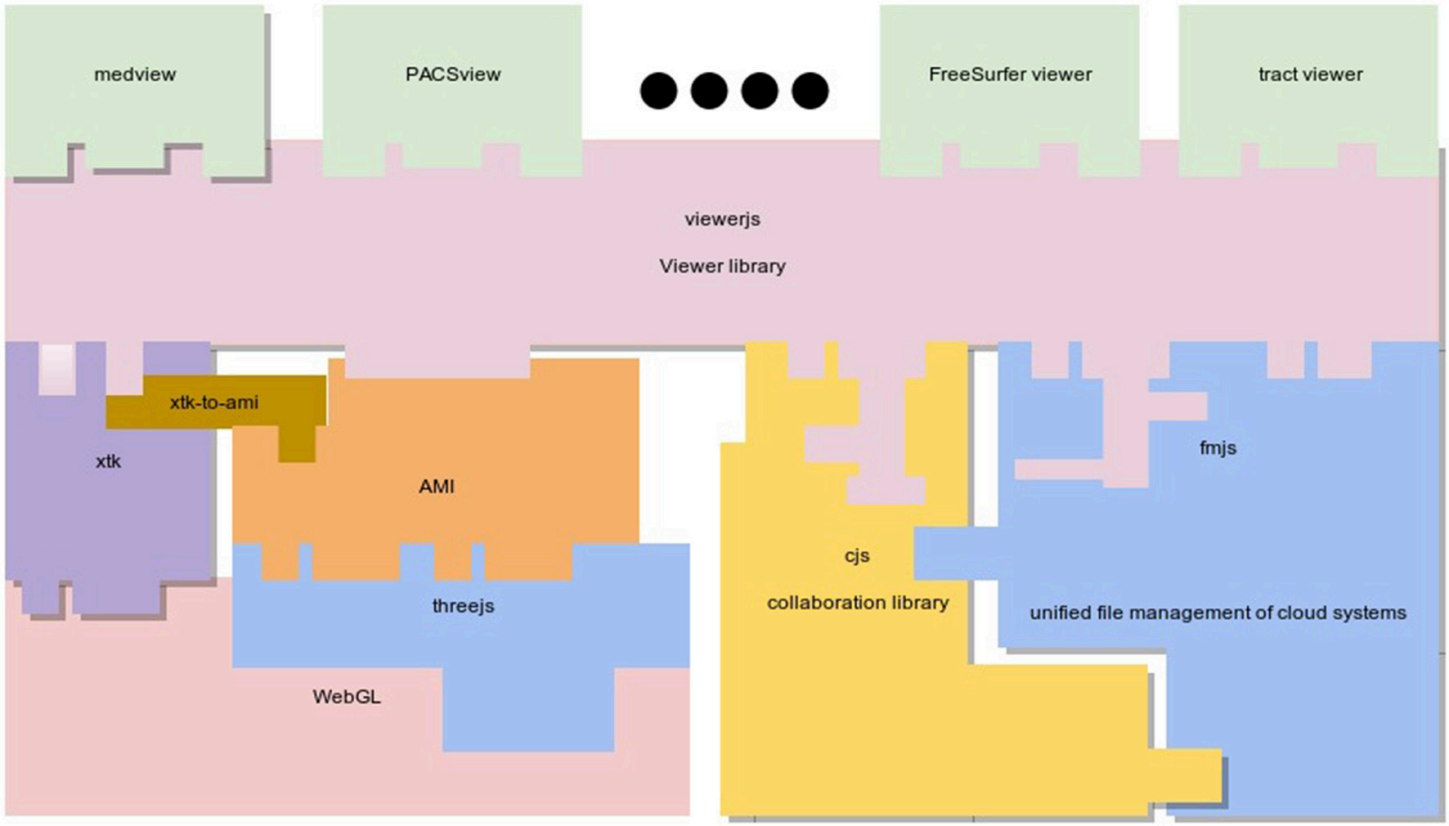
**The main logical components of ***Medview*****. The viewerjs library provides most of the services that an application such as *MedView* might require. Multiple viewers can quickly be constructed on viewerjs (for example a FreeSurfer surface viewer, a tractography viewer, etc). Internally, viewerjs uses low level graphical libraries (XTK and AMI), a real time collaboration library gcjs and a file management library fmjs. Note that the colors in the boxes are for ease of illustration and similarly colored boxes are not functionally related.

From an application programming perspective, an application like *MedView* is rather lightweight and most of the application visualization logic and behavior is provided by the viewerjs. This library is in turn reliant on several subcomponents—a low level visualization component (XTK), a collaboration component (gcjs), and a unified file management system (fmjs), see Figure 1.

Currently, the popular open-source XTK toolkit is used by viewerjs for low level graphic services. XTK is a fully client-side JavaScript library, which means it runs entirely in the web browser without requiring any special back-end infrastructure. It is built on top of the fast and well supported HTML5 Canvas 2D and WebGL 3D graphics' APIs by encapsulating functionality in an easy-to-use abstraction API for image visualization applications. This API is well documented with an extensive set of demos, lessons and practical code examples^9^. The library supports the most common neuroimage data formats including DICOM, NIFTI, MGH/MGZ, NRRD, VTK PolyData, Freesurfer meshes, STL and TrackVis as well as label maps, color tables and surface overlays (Haehn et al., 2014).

A successor to XTK called AMI^10^ is currently being developed. AMI features a highly efficient visualization pipeline and is built on top of the powerful threejs^11^ JavaScript library. Subsequent versions of viewerjs will transition to AMI for more powerful visualization operations. Importantly, the final application (like *MedView*) ideally does not need to call primitives at the rendering library directly.

The viewerjs library exposes a viewerjs.Viewer class. This class provides methods for easily embedding a neuroimage visualization object (VObj) within an HTML page. The viewerjs.Viewer constructor only requires as an input the Document Object Model (DOM) identifier of the HTML element on which the resultant VObj's HTML interface is inserted. The following code shows the simplicity of the method calls:


var view = new viewerjs.Viewer(divId);
view.init();
view.addData(imgFileArr);


The VObj can asynchronously load more than one neuroimage volume specified by the imgFileArr variable passed to the addData method. The imgFileArr is an array of custom file objects where each object entry has the following properties:

- url: String representing the file's URL/local path (required)
- file: HTML5 File object (optional but necessary when the files are sourced through a local file-picker or drop-zone)
- cloudId: String representing the file cloud identifier (optional but necessary when the files are sourced from a cloud storage service such as Google Drive)

Using the fmjs library, the VObj can load image data from diverse sources such as a remote service using the provided url, a local filesystem using the file property or the Google Drive storage service using the cloudId property. More data can be added to the viewer by repeatedly calling the addData method which creates a new thumbnail bar for each dataset (users can also interactively add more data by dragging files/folders onto the viewer—each drag/drop event will create a new floating thumbnail bar).

**Figure 2:**
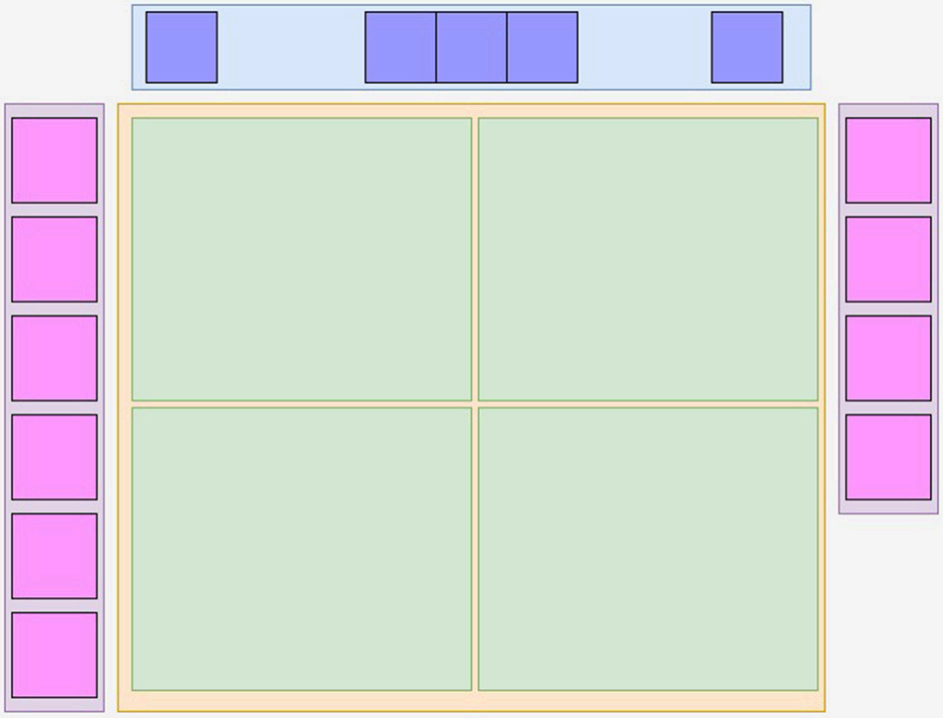
**The main logical components of ***Medview*****. At the top is a toolbar in blue provided by toolbarjs, and on the left and right are floating pink colored thumbnail bars containing the center image of a volume, provided by thbarjs. In the center is a yellow rboxjs container that houses one or more green rendererjs objects that provide image interactivity. An app such as *MedView* assembles these building blocks as it sees fit.

Using viewerjs, *MedView* constructs a graphical user interface (GUI) comprising the main functional components as shown in Figure 2. It contains a tool bar at top with action buttons (using toolbarjs^12^), a central neuroimage visualization square (provided by rboxjs^13^) that contains individual interactive visualizers, rendererjs^14^, and on each side, two floating thumbnail bars (thbarjs^15^) with an automatically generated snapshot image of the middle slice for each neuroimage volume. Currently the visualization objects rendererjs only provide cross-sectional slice rendering of the 3D datasets. These just use two types of XTK's objects that are closely associated, the X.Volume that contains the 3D volume data and the X.renderer2D that performs the actual rendering and visualization.

Up to four thumbnail images can be dragged and dropped from the thumbnail bar into the visualization square for simultaneous visualization of their corresponding volumes. This action also “removes" the volume from the thumbnail bar—closing a volume view returns the volume to its original thumbnail bar. The four-volume display limit is not programmatically imposed, but reflects a design choice to show multiple volumes without visually overwhelming the display. Only those volumes being visualized in the visualization square are kept in memory to reduce the possibility of out-of-memory crashes. Therefore, every time a thumbnail is dropped into the visualization square a new data loading is triggered from either the local filesystem or a remote service according to the location of the volume file. Once data is loaded locally in memory the rendering performs very rapidly as there is no network upload involved. For remote data, the speed of access is unavoidably limited by the network latency. Finally a volume can then be unloaded from the visualization square by dragging and dropping it back into the thumbnail bar. This modern and simple GUI allows users to quickly explore several 3D neuroimage volumes in a very intuitive manner.

### 2.3. Real-time synchronization

The client-side rendering approach adopted in *MedView* allows for a very responsive desktop-application-like visualization experience. Once a neuroimage volume has been loaded in the visualization square the user can interact with the data by manipulating the visualization through peripheral device controls and immediately sees the results of that interaction (e.g., moving the mouse to point to a different image location or rolling the mouse wheel to navigate across the volume slices by cross-section). The goal of the real-time collaboration is then to provide a way for simultaneous visualization of the same data by several collaborators working on remote computational entities and propagate the results of any user-data interaction to all the collaborators in real time. This requires a mechanism for sharing both the neuroimage data and the visualization state among collaborators.

The data sharing mechanism we provide here is based on two reusable JavaScript modules called fmjs and gcjs (see Figure 1 for library organization). The fmjs module is a file manager designed to provide a unified interface to common file operations on abstract filesystems such as the HTML5 sandboxed filesystem (currently available only in Chrome) and the Google Drive cloud storage service (GDrive). In particular, the exposed fmjs.GDriveFileManager class implements file uploading/downloading, file sharing and other operations on GDrive by leveraging the GDrive Representational State Transfer (REST) API^16^. The gcjs module on the other hand, exposes the gcjs.GDriveCollab class that reuses the fmjs.GDriveFileManager's functionality and the GDrive Realtime REST API^17^ to manage the real-time collaboration.

There are several advantages of choosing GDrive services and APIs for this client-side distributed application. First, they can be accessed directly from the client-side JavaScript code without requiring any back-end infrastructure. Second, there is no need for the heavy work of running our own data storage and real-time synchronization servers but instead we simply leverage the high availability and latency of the powerful GDrive servers. Third, conflict resolution is automatically handled when many collaborators are concurrently changing the visualization parameters. Last but not least, an OAuth 2.0-based^18^ mechanism for user authentication and authorization management is provided that allows for controlled access to collaborators' private data and identity.

The real-time collaboration is actually implemented by synchronizing the application data (visualization parameters) among collaborators using the GDrive Realtime Collaborative Data Model (RT-CDM) which is basically a hierarchy of collaborative objects with built-in synchronization among collaborators. When any data is modified in the RT-CDM or new application data is included they are automatically persisted and shared with all the collaborators^19^. The gcjs.GDriveCollab class provides methods to get and set the RT-CDM and five event listeners that can be dynamically overwritten on its object instances:

1. onConnect called by all connected instances just after a new instance connects to the collaboration session
2. onDataFilesShared called on all connected instances every time the collaboration owner has shared all the data files in its GDrive with a new collaborator
3. onCollabObjChanged called on all connected instances every time the RT-CDM is updated by any remote collaborator
4. onNewChatMessage called by all connected instances everytime a new chat message is received from a remote collaborator
5. onDisconnect called by all connected instances everytime a remote collaborator disconnects

A gcjs.GDriveCollab instance can allow any client-side JavasScript application the ability to participate in a real-time collaboration session through these methods and custom event listeners.

Indeed the viewerjs.Viewer constructor of the previous section can accept a gcjs.GDriveCollab object as an optional second parameter to enable the collaboration among remote visualizations. The resultant VObj delegates the synchronization of the data describing the visualization state on that object. These data are mainly comprised of a small JavaScript Object Notation (JSON) object with a few numeric and string properties describing the state of the XTK's X.Volume and X.renderer2D objects currently instantiated in the VObj's visualization square. This information is available thanks to the XTK API exposing the state of its graphics objects which can easily be modified not only through device controls (e.g., mouse wheel) but also programmatically with the corresponding automatic visualization update. Thus, by keeping the lightweight JSON object describing the state of the graphics objects in sync among remote instances the actual visualizations are also automatically synchronized.

A collaboration session starts when one user clicks the button “Start collaboration” in the VObj's toolbar. A new modal window pops up to let the user decide if she wants to start a new collaboration session as the collaboration owner or instead join an existing collaboration session. Either choice triggers Google's authorization flow so that the user can log into their Google account and authorize the VObj to access its GDrive space. After successful authorization a floating chat window with a collaboration session identifier (id) shows up on top of the VObj's GUI. This id (similar to a chat room id) can then be sent to other users by email or any other on-line messaging system so they can use it to connect to the current collaboration session through their local VObj. The actual neuroimage data files (all the volumes corresponding to the thumbnail images in the thumbnail bar) are uploaded to the collaboration owner's GDrive. However, if any neuroimage volume is comprised of many Digital Imaging and Communications in Medicine (DICOM) files then they are first concatenated into a small number of compressed (zip) files before uploading to GDrive. This is done mainly to reduce the number of required HTTP connections and network bandwidth usage but it is also useful to reduce the number of automatic notification emails received by the other collaborators when these files are shared with them in GDrive. Unlike Slice:Drop, the uploaded files are not publicly shared with the whole Internet. They are only automatically shared with the other authenticated collaborators on demand when they connect to the collaboration session. At that point their VObj instance will then automatically download a copy of the data files from GDrive for their local rendering and visualization.

### 2.4. Real-time implications

The real-time model described here does have some important implications to consider. In order to allow for responsive client behavior, each participating client needs a complete copy of the image data to render locally. The delay in joining a collaborative session is thus a strong function of network bandwidth between the client and the GDrive servers. Relatively long delays may be experienced on slow connections, especially if many (or large) data sets are being shared.

Real-time collaboration is best intended for single image (or volume image) cases and not really multiple image sets concurrently. Moreover, despite the startup delay in sharing multiple image sets, the viewing experience is limited by the memory available to a browser. The operation of the technology may be unworkable in cases where limited memory and/or bandwidth environments exist.

### 2.5. Development and build system

We adopted a modular software development strategy that allows for separation of concerns, improves code reusability and facilitates application development and maintenance.

The source files for each JavaScript module and its testing code are kept separate from other modules' source files by hosting them on their own independent Github repositories. Automatic fetching of file dependencies between modules is carried out using Bower^20^ which is a popular package manager that can recursively download dependency source files from Github repositories and other sources. Some of the external dependencies required by the developed modules and automatically fetched through Bower include jQuery^21^ and jQuery UI^22^ employed in the implementation of the interactive GUI, dicomParser^23^ to extract meta information from DICOM files and JSZip^24^ for file concatenation and compression. The logical dependencies between modules are then solved using RequireJS^25^, a commonly used JavaScript loader, also available through Bower, that can asynchronously load into the browser any module required by the currently executing client-side code. Finally, all the typical JavaScript development and building tasks such as code hinting, testing, concatenation, minification and generation of the production application files are automatically managed by Grunt^26^ which is a widely used JavaScript task runner based on the Node platform^27^.

As an integrative example the *MedView* app's source code can be downloaded to a local development machine by cloning its Github repository^28^. The developer can then change the working directory to the created local git repository and follow the detailed instructions in the README file to build and test a local version of the web application. The app's source code basically showcases the usage of the proposed JavaScript modules whose source codes are in turn fetched within the bower components directory at the root of the repository.

### 2.6. Comparison with slice:drop

*MedView* can be thought of as a logical successor to our previous work, Slice:Drop (Haehn (2013)). Several key technological differences exist between this work and Slice:Drop. Perhaps most importantly from a software development perspective, Slice:Drop was more of a prototype and less of a fully engineered/designed application. Its internal structure was monolithic and not modularized in terms of functionality, unlike *MedView* which has a reusable modular library design. For example, in Slice:Drop the data push and pull to the Dropbox servers (for collaboration) is an inherent part of the code and not at all easily extractable for use elsewhere, while in *MedView* all push/pull is modularized in the reusable gcjs library which can effectively be used by any application.

*MedView* also uses OAuth 2.0 for its user authentication and authorization management which allows a fine grain of access control to uploaded data. In Slice:Drop files uploaded to Dropbox have no authorization control and are fully publicly accessible.

In terms of collaboration, *MedView* has an integrated chat client, while in Slice:Drop the chat was an external application—practically in *MedView* the chat experience feels more integrated into the system. Most importantly, *MedView* offers a shared cursor among collaborators in their viewers, which is not a feature of Slice:Drop.

Finally, in *MedView* multiple image volumes can be shared in a collaboration session, unlike in Slice:Drop wherein only one image volume can be shared collaboratively.

## 3. Results

The JavaScript modules implementing the proposed technological solution have been instantiated in the development of a distributed collaborative neuroimage visualization application called *MedView* which basically showcases the VObj usage and the way it can easily be embedded in any web application.

**Figure 3:**
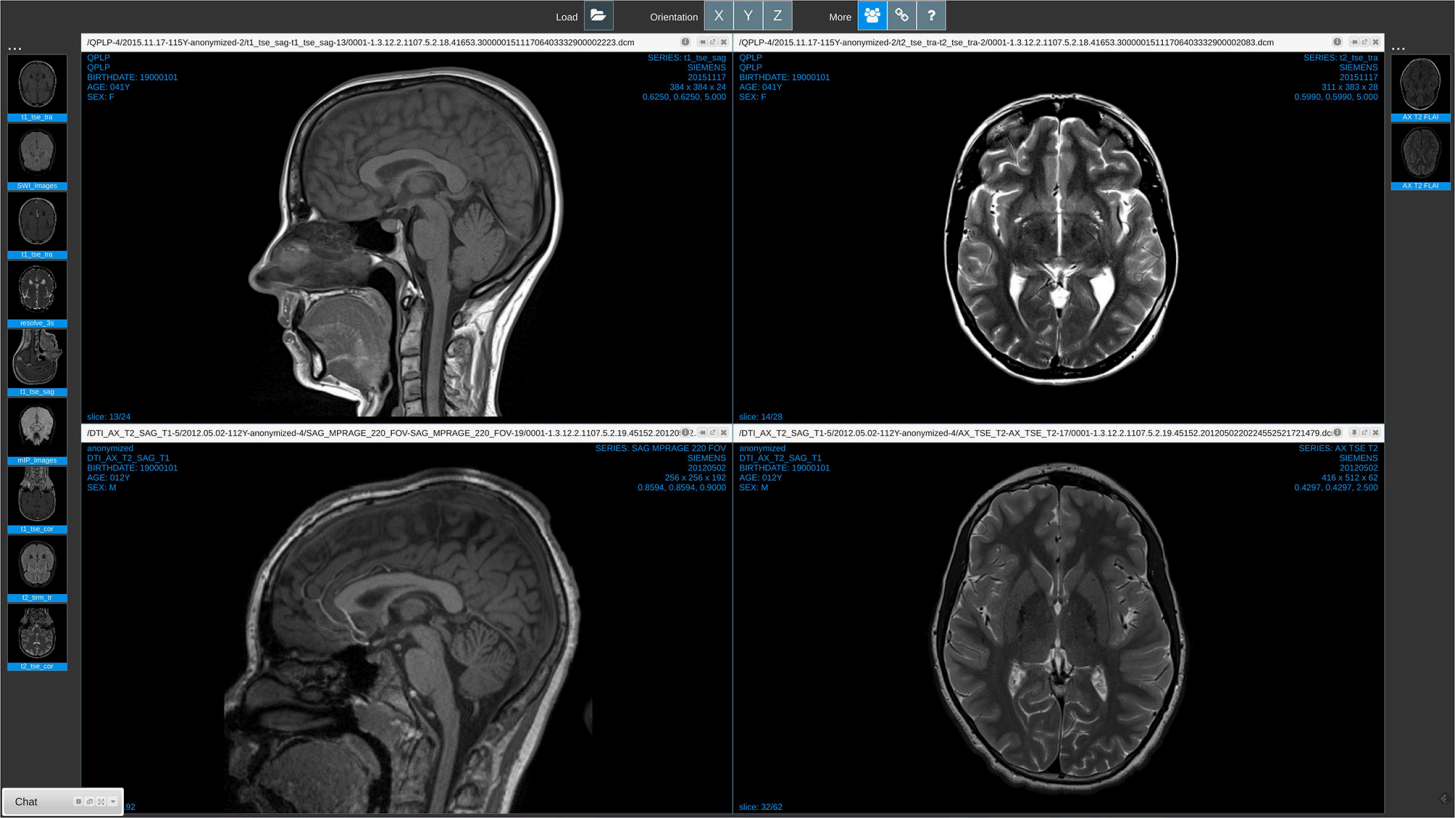
*****Medview*** on a workstation**. A screenshot of a session running on a Linux workstation in the Chrome Browser. The collaboration icon in the toolbar (third from left) is active, and on the bottom left a minimized chat window provides visual indication that this session is currently linked to collaborators.

*Medview* is designed as a simple, robust, and multi-device web-app. By simply pointing a browser at the *MedView* URL^29^ almost any device can view and interact with most medical image formats. For example, on a Linux host session running Google Chrome in Figure 3, the user opened the standard graphical desktop filebrowser, and navigated to a directory containing medical image files. The parent directory was simply dragged and dropped into the main *MedView* window. The thumbnail bar on left was generated. In this instance, the image volumes were all NifTI data formats. The volumes were read into the browser, the center slice in the acquisition direction determined, and that slice was rendered in the thumbnail representation. A second directory, itself containing nested sub-directories of DICOM data was also dragged into the browser. This created a new, second thumbnail bar (on right) and again the center DICOM of each series uploaded is shown as representative of that volume. Each action of dragging and dropping from the host's filesystem into the browser, triggers the creation of a new thumbnail bar. The user can drag these bars and position them on either the left or the right of the screen.

Note that at time of writing only Google Chrome supports recursive directory processing. In other browsers, such as FireFox, Safari, and Microsoft Edge, actual volume files have to be explicitly selected and dragged/dropped.

**Figure 4:**
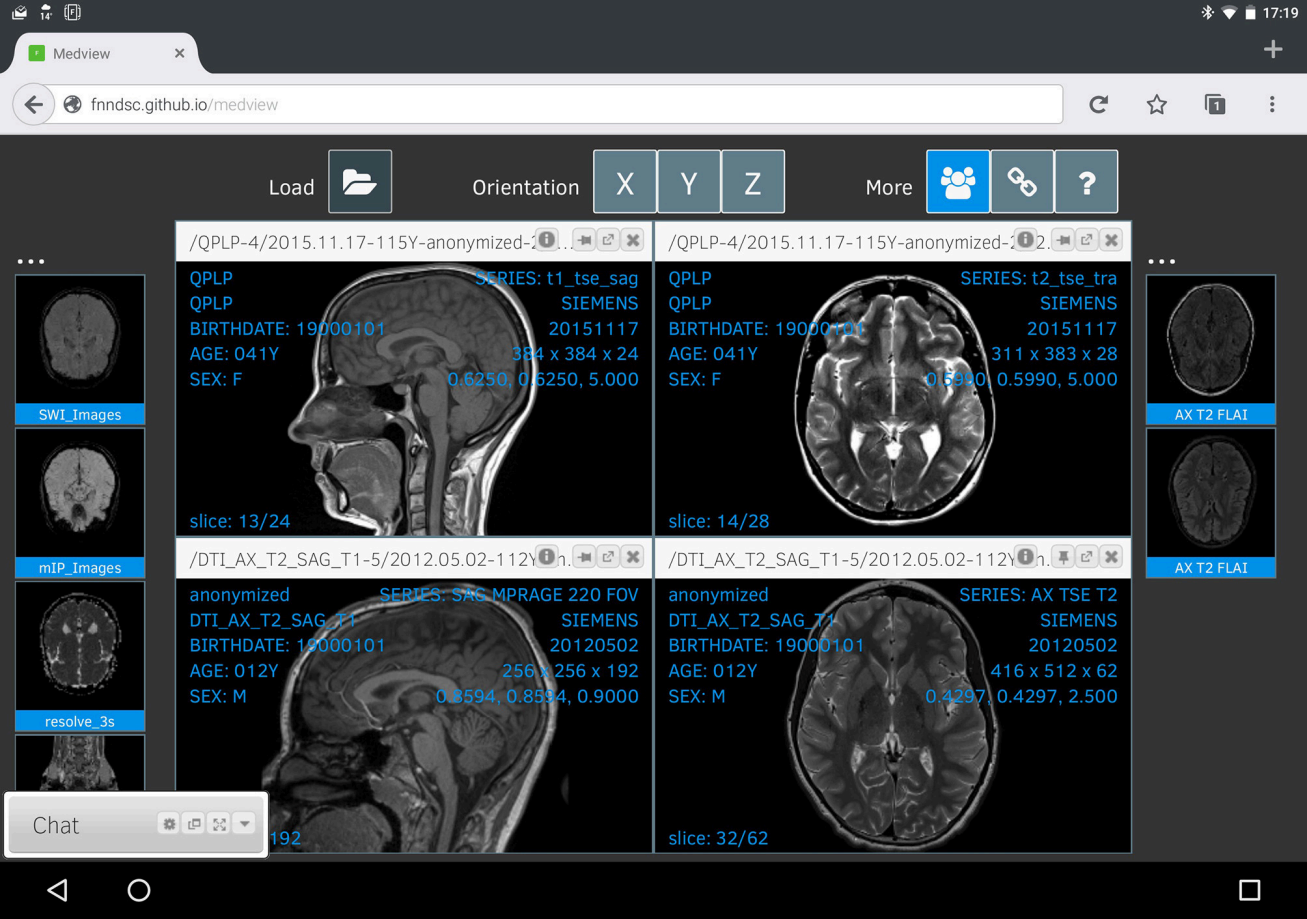
*****Medview*** on a tablet**. A screenshot of a collaborative session, captured on an Android tablet running Firefox. Any changes to the visual state of this session are immediately shared with linked collaborators, and vice-versa.

Finally, Figure 4 shows a linked collaborative session as seen from an Android tablet running FireFox. Due to the constrained resolution, the main viewer windows are smaller and there is some font interference (which will be addressed in future updates).

The order of the thumbnails in each collaborative session might be unique to that session itself, but the main render displays are intimately linked for each participant. While not explicitly shown, a shared pointer-cursor is also available that appears on the same location on all linked images (with the mouse over a specific image volume press the SHIFT key and then move the mouse to the desired location). In this manner, any collaborator can explicitly highlight an exact pixel on a given image and have that information communicated between all linked sessions.

## 4. Discussion

We have presented a novel web-based software solution to the problem of implementing efficient real-time collaborative neuroimage visualization. A client-side rendering and visualization approach based on the XTK toolkit for “low level" graphical rendering in JavaScript and the fmjs and gcjs libraries that provide for highly responsive real-time collaboration. Once a neuroimage volume has been loaded in the browser the results of subsequent user-data interactions are seen by the local user instantaneously. Any linked collaborators might experience a slight delay as the small real-time state object is propagated across the network using the Google GDrive RT-CDM. This conveys just enough information to describe the current visualization state, effectively minimizing the amount of data that needs to be serialized and distributed over the network. This is in contrast with previous server-side rendering solutions in which both local and remote users can experience low-responsive visualization interactions due to the continuous and heavy network traffic between the server and the connected clients. In addition the server-side rendering approach doesn't scale well as the number of concurrent users increases as the server can quickly become overwhelmed by the heavy data processing and network usage (Kaspar et al., 2013).

The open-source object-oriented JavaScript modules are highly reusable and can be easily integrated in existing or new web-based applications like *MedView*. These types of web-based distributed applications are very appealing as they can be delivered to users over the web without requiring the installation of any additional software or browser plug-ins. This not only enhances accessibility and usability for the end-users but automatically provides them with the latest application updates without requiring any technological skills or administrator-level access to their computing devices.

Moreover, given the rise of mobile devices and technology, as well as powerful mobile-versions of mainstream browsers, tablets and smartphone devices can fully participate in this technology without requiring mobile-specific versions of the apps and libraries. Javascript in this case is a truly universal language and technology.

Several limitations to the technology and solution presented in this paper do exist. Firstly, this work is intended for research use only—the security model and code do not purport to be ready for clinical certification. Furthermore, though the solution presented here is completely opensource, we do rely on Google services in the background to provide the “plumbing" that enables the real-time collaboration. This is deemed acceptable, however, due to the ubiquity of Google services and the off-the-shelf leveraging of existing, powerful solutions.

Given the intrinsic needs for cooperative work in the medical and neuroimage research, clinical, and education fields, web-based collaborative image visualization systems are becoming more and more important and they can only reach their full potential by including real-time interactive collaboration features. The technological solution presented in this paper provides a rapid and practical way of implementing those requirements. Overall, this real-time interactive collaboration over the Internet is quite feasible using the latest advances in core web technologies and web-based distributed application development (Flanagan, 2011; Richardson et al., 2013). Future work in *MedView* will focus on improved mobile experience and the development of new JavaScript modules to enable in-app on-line video calls based on the WebRTC standard^30^, as well as continual improvements in the core graphical rendering components.

## Author contributions

JB: Main coding of MedView. NR: Coding of XTK library. RG: Deployment of medview in clinical context. SP: Design/UI. SM: Deployment of medview in clinical context. RR: Design feedback. PG: UI/UIX. RP: Architecture/lead.

### Conflict of interest statement

The authors declare that the research was conducted in the absence of any commercial or financial relationships that could be construed as a potential conflict of interest. The reviewer ZRQ and handling Editor declared their shared affiliation, and the handling Editor states that the process nevertheless met the standards of a fair and objective review.

## Footnotes

^1^http://fnndsc.babymri.org.

^2^http://goxtk.com.

^3^http://slicedrop.com.

^4^https://github.com/FNNDSC/viewerjs.

^5^https://github.com/FNNDSC/fmjs.

^6^https://github.com/FNNDSC/gcjs.

^7^https://github.com/FNNDSC.

^8^https://fnndsc.github.io/medview.

^9^https://github.com/xtk/X.

^10^https://github.com/FNNDSC/AMI.

^11^https://threejs.org.

^12^https://github.com/FNNDSC/toolbarjs.

^13^https://github.com/FNNDSC/rboxjs.

^14^https://github.com/FNNDSC/rendererjs.

^15^https://github.com/FNNDSC/thbarjs.

^16^https://developers.google.com/drive/v2/reference/.

^17^https://developers.google.com/google-apps/realtime/.

^18^https://developers.google.com/identity/protocols/OAuth2UserAgent.

^19^https://developers.google.com/google-apps/realtime/build-model.

^20^http://bower.io/.

^21^https://jquery.com/.

^22^https://jqueryui.com/.

^23^https://github.com/chafey/dicomParser.

^24^https://stuk.github.io/jszip/.

^25^http://requirejs.org/.

^26^http://gruntjs.com/.

^27^https://nodejs.org/.

^28^https://github.com/FNNDSC/medview.

^29^http://fnndsc.github.io/medview.

^30^http://www.webrtc.org.

## References

[B1] Anupam V. Bajaj C. Schikore D. Schikore M. (1994). Distributed and collaborative visualization. Computer 27, 37–43. 10.1109/2.299409

[B2] Coleman J. D. Klement E. Savchenko A. Goettsch A. (1997). Teleinvivo: a novel telemedical application for collaborative volume visualization, in Proceedings of the Fourth ACM International Conference on Multimedia, (Boston, MA: Fetal-Neonate DevCenter), 445–446.

[B3] Eckersley P. Egan G. F. De Schutter E. Yiyuan T. Novak M. Sebesta V.. (2003). Neuroscience data and tool sharing. Neuroinformatics 1, 149–165. 10.1007/s12021-003-0002-1 15046238

[B4] Flanagan D. (2011). JavaScript: The Definitive Guide: Activate Your Web Pages. Sebastopol, CA: O'Reilly Media, Inc.

[B5] Forslund D. W. George J. E. Gavrilov E. M. Staab T. Weymouth T. E. Kotha S. (1998). Telemed: development of a java/corba-based virtual electronic medical record, in Medical Technology Symposium, 1998. Proceedings. Pacific, (IEEE), 16–19. 10.1109/PACMED.1998.767876

[B6] Forslund D. W. Phillips R. L. Kilman D. G. Cook J. L. (1996). Telemed: a working distributed virtual patient record system, in Proceedings of the AMIA Annual Fall Symposium (Washington, DC: American Medical Informatics Association), 990.

[B7] Gokhale A. Kumar B. Sahuguet A. (2002). Reinventing the wheel? corba vs. web services, in Proceedings of International World Wide Web Conference (Honolulu, HI).

[B8] Haehn D. (2013). Slice: drop: collaborative medical imaging in the browser, in ACM SIGGRAPH 2013 Computer Animation Festival (Anaheim, CA: ACM).

[B9] Haehn D. Rannou N. Ahtam B. Grant E. Pienaar R. (2014). Neuroimaging in the browser using the x toolkit, in Frontiers in Neuroinformatics Conference Abstract: 5th INCF Congress of Neuroinformatics (Munich).

[B10] Henning M. (2006). The rise and fall of corba. Queue 4, 28–34. 10.1145/1142031.1142044

[B11] Kaspar M. Parsad N. M. Silverstein J. C. (2010). Cowebviz: interactive collaborative sharing of 3d stereoscopic visualization among browsers with no added software, in Proceedings of the 1st ACM International Health Informatics Symposium (Arlington, VA: ACM), 809–816.

[B12] Kaspar M. Parsad N. M. Silverstein J. C. (2013). An optimized web-based approach for collaborative stereoscopic medical visualization. J. Am. Med. Inform. Assoc. 20, 535–543. 10.1136/amiajnl-2012-001057 23048008 PMC3628048

[B13] Manssour I. H. Dal Sasso Freitas C. M. (2000). Collaborative visualization in medicine, in WSCG '2000: Conference proceeding: The 8th International Conference in Central Europe on Computers Graphics, Visualization and Interactive Digital Media '2000 in cooperation with EUROGRAPHICS and IFIP WG 5.10: University of West Bohemia (Plzen), 266–273.

[B14] Millan J. Yunda L. (2014). An open-access web-based medical image atlas for collaborative medical image sharing, processing, web semantic searching and analysis with uses in medical training, research and second opinion of cases. Nova 12, 143–150.

[B15] Mwalongo F. Krone M. Reina G. Ertl T. (2016). State-of-the-art report in web-based visualization. Comput. Graph. Forum 35, 553–575. 10.1111/cgf.12929

[B16] Pienaar R. Rannou N. Bernal J. Hahn D. Grant P. E. (2015). ChRIS – A web-based neuroimaging and informatics system for collecting, organizing, processing, visualizing and sharing of medical data, Conference proceedings: Annual International Conference of the IEEE Engineering in Medicine and Biology Society. IEEE Engineering in Medicine and Biology Society. Annual Conference (Milan, IL). 10.1109/EMBC.2015.7318336 26736236

[B17] Richardson L. Amundsen M. Ruby S. (2013). RESTful Web APIs. Sebastopol, CA: O'Reilly Media, Inc.

[B18] Sherif T. Kassis N. Rousseau M.-É. Adalat R. Evans A. C. (2015). Brainbrowser: distributed, web-based neurological data visualization. Front. Neuroinform. 8:89. 10.3389/fninf.2014.00089 25628562 PMC4292582

[B19] Sherif T. Rioux P. Rousseau M.-E. Kassis N. Beck N. Adalat R.. (2014). Cbrain: a web-based, distributed computing platform for collaborative neuroimaging research. Front. Neuroinform. 8:54. 10.3389/fninf.2014.00054 24904400 PMC4033081

[B20] Wood D. King M. Landis D. Courtney W. Wang R. Kelly R.. (2014). Harnessing modern web application technology to create intuitive and efficient data visualization and sharing tools. Front. Neuroinform. 8:71. 10.3389/fninf.2014.00071 25206330 PMC4144441

